# Anion–cation contrast of small molecule solvation in salt solutions†

**DOI:** 10.1039/d1cp04129k

**Published:** 2022-01-04

**Authors:** Stefan Hervø-Hansen, Jan Heyda, Mikael Lund, Nobuyuki Matubayasi

**Affiliations:** Division of Theoretical Chemistry, Department of Chemistry, Lund University Lund SE 221 00 Sweden stefan.hervo_hansen@teokem.lu.se +46 46 222 8251; Department of Physical Chemistry, University of Chemistry and Technology Prague CZ-16628 Czech Republic heydaj@vscht.cz +420 22044 4297; Division of Theoretical Chemistry, Department of Chemistry, Lund University Lund SE 221 00 Sweden mikael.lund@teokem.lu.se +46 46 222 3167; Lund Institute for Advanced Neutron and X-ray Science (LINXS), Lund University Lund Sweden; Division of Chemical Engineering, Graduate School of Engineering Science, Osaka University Toyonaka Osaka 560-8531 Japan nobuyuki@cheng.es.osaka-u.ac.jp +81-6-6850-6343 +81-6-6850-6565

## Abstract

The contributions from anions and cations from salt are inseparable in their perturbation of molecular systems by experimental and computational methods, rendering it difficult to dissect the effects exerted by the anions and cations individually. Here we investigate the solvation of a small molecule, caffeine, and its perturbation by monovalent salts from various parts of the Hofmeister series. Using molecular dynamics and the energy-representation theory of solvation, we estimate the solvation free energy of caffeine and decompose it into the contributions from anions, cations, and water. We also decompose the contributions arising from the solute–solvent and solute-ions interactions and that from excluded volume, enabling us to pin-point the mechanism of salt. Anions and cations revealed high contrast in their perturbation of caffeine solvation, with the cations *salting-in* caffeine *via* binding to the polar ketone groups, while the anions were found to be *salting-out via* perturbations of water. In agreement with previous findings, the perturbation by salt is mostly anion dependent, with the magnitude of the excluded-volume effect found to be the governing mechanism. The free-energy decomposition as conducted in the present work can be useful to understand ion-specific effects and the associated Hofmeister series.

## Abstract 2

Dissected contributions from anion, cation, and water in ion-specific effects on caffeine solvation.

## Graphical abstract

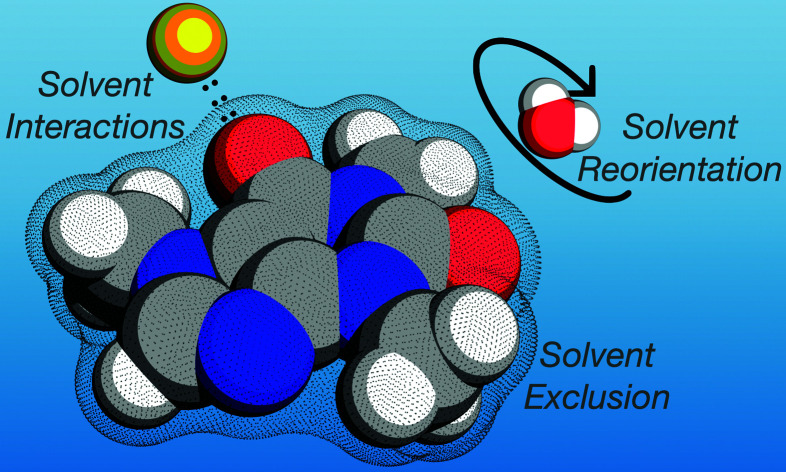

## 1 Introduction

Caffeine is probably the most consumed psychoactive drug worldwide,^1^ most commonly found in coffee, tea, and energy drinks. Despite caffeine typically being characterised as a bitter taste stimulant,^2^ the caffeine-containing beverages are paradoxically considered by many a great joy. Therefore the process of coffee brewing has undergone tremendous development and experimentation in order to obtain correct amounts of caffeine by extraction from solid to the aqueous phase in the goal of creating the perfect cup of coffee.^3–5^ Consequently, it is desirable to understand the physical and chemical properties of caffeine to optimise and understand processes in which caffeine is involved, including drink brewing such as coffee and tea, medicine, and other industrial, pharmaceutical, and biological proposes.

Due to the previously mentioned reasons, and due to caffeine also possessing a chemical structure related to the purine nucleobases of DNA and RNA, the physical properties of caffeine has been exhaustively investigated by a great variety of methodologies, including experimental, computational, and theoretical methods. In particular it is known that caffeine is surprisingly soluble in polar solvents with a preference for chloroform over water,^6^ while only sparsely soluble in non-polar organic solvents, due to caffeine's molecular structure being heterogeneous in terms of polarity. Additionally, in neutron scattering experiments, caffeine has also been found to possess a self-association equilibrium, forming highly ordered oligomers characterised by the face-to-face stacking of the xanthine motif,^7–10^ similar to the stacking of the nucleobases found in DNA and RNA.^11^ However, the formation of larger aggregates has also been reported, in which the oligomers are also branched at the methyl groups.^12^ The mentioned equilibria; the partitioning of caffeine in the aqueous and organic phases, and the self-association of caffeine are all subject to modulation by osmolytes such as sugars,^13–15^ and salts.^14,16,17^

The perturbation by salt is typically qualified using the historic terms *salting-in* and *salting-out* characterising the salt effect either to increase or decrease the solubility of solute in a given solvent respectively. However, towards the end of the 19th century Franz Hofmeister discovered an ordering of anions and cations in their ability to modulate the solubility of hen egg-white albumin, thus quantifying the effect of salt.^18,19^ This ordering, today known as the Hofmeister series of ions, is one of the fundamental discoveries within the field of ion-specific effects. It has later been found that the Hofmeister series are universal and more generally applicable in the perturbation of equilibria, ranging from solubility and structural stability of various proteins to the solubility and aggregation of small molecules like caffeine. With the finding of ion-specific effects, effort has been put into gaining mechanistic insight underlying the Hofmeister series for various systems. Early theories focused on the categorisation of ions into two different groups: kosmotrope ions (water-structure makers) which are characterised by being well hydrated, and chaotrope ions (water-structure breakers) characterised by poor hydration. These theories, however, may not be in agreement with the observations from modern spectroscopic experiments and molecular dynamics simulations, in which only local effect of ions on water is observed.^20,21^ Yet, despite the recent progress, the molecular mechanism of the Hofmeister series is still not complete.^22,23^ The two most common mechanistic models to date are:^22–26^ (I) The indirect mechanism of action, in which ions interact with water to modulate the solute-water interacions; (II) the direct mechanism, in which ions interact with the solute.

For caffeine, the direct and indirect mechanism models have been applied to explain the perturbation of anions on the partitioning between aqueous and cyclohexane phases. The weakly hydrated anions bound to the methyl groups and aromatic heteroatomic ring exert *salting-in* effects *via* the direct mechanism, while the strongly hydrated anions are excluded to *salt-out* caffeine *via* the indirect mechanism.^16^ The binding of weakly hydrated anion was also investigated by NMR spectroscopy obtaining dissociation constants and the site of association. It was revealed that the former type of anions decreases the formation of caffeine oligomers, while the latter promotes the oligomerisation.^17^ Through the analyses of Kirkwood–Buff integrals Shimizu found that the direct ion–caffeine interactions present a primary contribution to the caffeine dimerization equilibrium.^14^ Furthermore, the importance of the investigation of water mediated ion–caffeine's interactions was proposed.^14^

Despite the knowledge gained about caffeine's behaviour in the presence of salt, the majority of focus has been attributed to the anion effects or co-solvent as a whole, while the analysis of the cation effect remains incomplete. Consequently we here investigate the effect exerted by anion and cation separately on the solubility of caffeine in water. Utilising molecular dynamics and free energy calculations, we unveil the contrast in the contributions from anion and cation to the solvation free energy of caffeine, illustrating anions to *salt-out* while cations *salt-in*. The excluded-volume effect will also be revealed as the factor to govern the ion-specific effects. The free energy method utilised is the energy-representation theory of solvation.^27–29^ As a notable feature of the theory, it offers a straightforward route to species decomposition and energy-domain decomposition. The method still offers a comparable accuracy as other state of the art free energy methods, like Bennett's acceptance ratio method, for the solvation of small compounds in aqueous solutions.^30^

## 2 Method

All-atom molecular dynamics (MD) simulations were conducted using the openMM (7.4.0) software package^31^ modded with the OpenMMTools (0.18.3)^32^ and ParmEd (3.2.0)^33^ packages. For caffeine, a GROMOS (ffGF53a6) derived Kirkwood–Buff force field with adjustment to the partial charges and geometrical parameters was utilised, which has previously been able to reproduce experimental solvation properties such as solvation enthalpy and number of solute–solvent hydrogen bonds.^7,8^ The caffeine system was simulated with the SPC/E model of water^34^ in combination with optimised ion parameters for halide anions and alkali cations.^35^ The isothermal–isobaric (*NPT*) ensemble was sampled through a geodesic symmetric Langevin Velocity-Verlet integrator^36^ with a temperature of 298.15 K, 3 geodesic drift steps at each integration step, a collision rate of 1.0 ps^−1^, and integration time step of 2 fs, and pressure regulation through an isotropic Monte Carlo barostat^37,38^ at 1 bar pressure, with volume move attempts every 0.05 ps.

For each system, 25 different initial configurations were created using Packmol (18.169)^39^ with the system geometry being cubic with a cell length of 32.5 Å containing 1000 water molecules and 0, 9 (∼0.5 M), 18 (∼1.0 M), 27 (∼1.5 M), and 36 (∼2.0 M) salt pairs. All initial configurations were first minimised using the limited-memory BFGS optimisation algorithm^40^ with a tolerance of 1 kJ mol^−1^ and a maximum of 5 × 10^5^ iterations. The atoms from the minimised configurations were assigned Maxwell–Boltzmann distributed velocities at 298.15 K followed by equilibration of 0.1 ns. To calculate the solvation free energy of caffeine, each system was simulated in the presence of caffeine (solution system), in the absence of caffeine (reference system), and for an isolated caffeine in vacuum. The reference system (water and salt) was simulated for a total of 10 ns with collection of configurations for statistical evaluation every 1 ps, while the solution system (water, caffeine, and salt) was simulated for a total of 50 ns with a sampling interval of 0.1 ps. The evaluation of electrostatic potential energies and forces were conducted using the Particle Mesh Ewald (PME) summation^41^ method with fifth order cubic interpolation, Ewald error tolerance of 10^−5^, and a real-space cutoff of 1.2 nm. The Lennard-Jones interactions were subject to switching functions on the form of a fifth order potential, with a switching range of 1.0–1.2 nm.

To include the effect of flexibility of solute in the transfer of caffeine from vacuum to solvent and salt, caffeine was simulated in vacuum in the canonical ensemble (*NVT*) at 298.15 K by Langevin dynamics, using the previously mentioned geodesic symmetric Langevin Velocity-Verlet integrator, with the difference being the usage of an integration time step of 1 fs and centre of mass motion removal. The Lennard-Jones conditions were identical to those for the solution and reference systems while the electrostatic interactions were handled in their generic 1/*r* forms, without a cutoff.^42^ The vacuum simulation was run for 50 ns with sampling of structural configurations every 0.01 ps.

The 25 trajectories for each system were analysed independently for the estimation of standard errors unless otherwise noted. Structural properties such as radial distribution functions were obtained using MDtraj (1.9.3).^43^ The solvation free energy was estimated using energy-representation theory of solvation^27–29^ (see Appendix A) as incorporated in the software ERmod (0.3.7).^42^ At the calculation of solvation free energies, long range dispersion correction was added to capture the contribution from Lennard-Jones interactions beyond the cutoff distance.^44^

The robustness of our theoretical approach was verified by repeating the above calculations using the OPLS force field for caffeine and ions accompanied by SPC/E water.^45,46^ These results are presented in ESI.†

## 3 Results and discussion

### 3.1 Quantifying salts' effects on the solvation of caffeine: Setschenow coefficients

For small, neutral solutes the empirical Setschenow equation provides a linear relationship between the salt concentration *c*_s_ and the logarithmic ratio of the solubility of a solute in the presence and absence of salt,^47^ with the proportionality constant being the Setschenow coefficient *k*_s_1
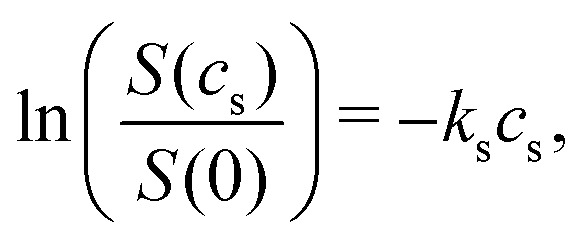
where *S*(0) and *S*(*c*_s_) are the solubilities of solute in pure water and an electrolyte solution containing the salt concentration *c*_s_, respectively. Historically the Setschenow equation utilised the decimal-logarithm, however, for simplicity we adopt the natural logarithm. The sign of the Setschenow coefficient *k*_s_ qualifies a salt's attribute to either be *salting-in* (*k*_s_ < 0), *i.e.* increasing the solubility of the solute by addition of salt or to be *salting-out* (*k*_s_ > 0), *i.e.*, decreasing the solubility.

**Fig. 1:**
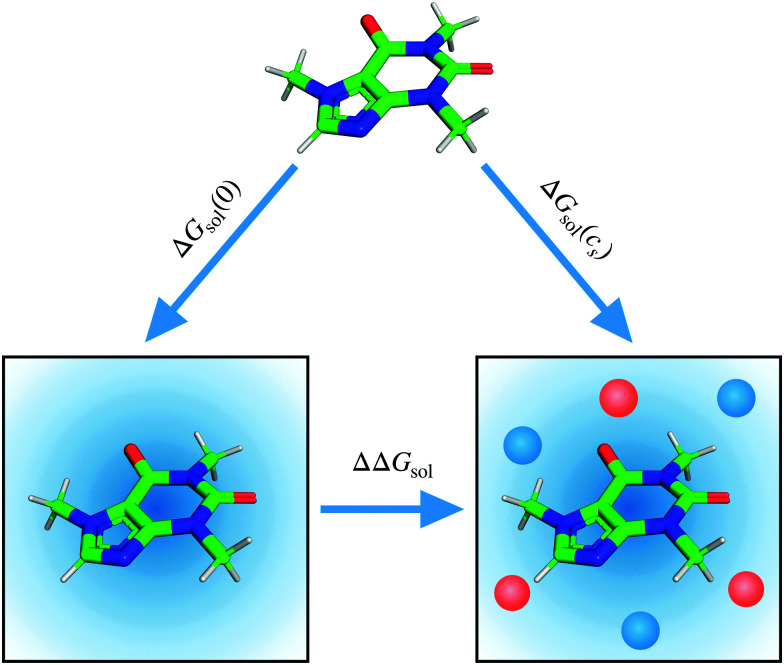
Thermodynamic cycle for the calculation of the change in the solvation free energy of caffeine upon addition of salt (lower horizontal axis). The diagonal axes of the cycle refer to the solvation of caffeine from vacuum to solvent and potential co-solvent.

The ratio of the solubilities at equilibrium is related to the difference in the solvation free energy upon addition of co-solvent (salt) ΔΔ*G*_sol_^48^2
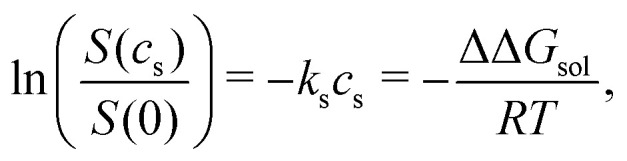
where *R* is the gas constant and *T* is the temperature. In eqn (2) ΔΔ*G*_sol_ is the difference in the solvation free energy Δ*G*_sol_, which is the transfer free energy from vacuum to the solution of interest and is equal to the free energy of turning on the interactions between the solute and the solvent (and co-solvent) (see Appendix A for a more detailed account). ΔΔ*G*_sol_ can be calculated as the difference in Δ*G*_sol_ for the solute being transferred from vacuum to an aqueous solution containing a finite concentration of salt and from vacuum to pure water due to the cancellation of the free energy of the vacuum state (Fig. 1):

3 ΔΔ*G*_sol_ = Δ*G*_sol_(*c*_s_) − Δ*G*_sol_(0).

**Fig. 2:**
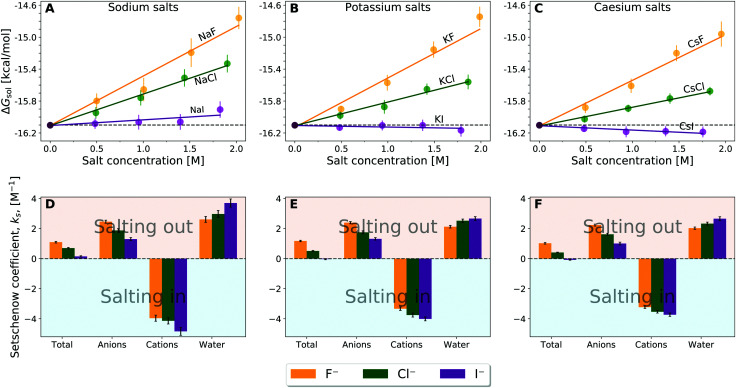
Solvation free energy, Δ*G*_sol_, of caffeine as a function of salt concentration (*top*) (A) sodium salts, (B) potassium salts, and (C) caesium salts with the colour differentiating the corresponding anion as donated by the legend. Setschenow coefficient, *k*_s_, and the contributions from the solvent species (*cf.*eqn (6)) namely anions, cations, and water (*bottom*) for (D) sodium salt solutions, (E) potassium salt solutions, and (F) caesium salt solutions. The anion, cation, and water contributions in (D–F) correspond to the first, second, and third terms of eqn (6). The self-energy correction was found not to vary with increasing salt concentration. The error bars shown report the 95% confidence interval with the errors for the Setschenow coefficients being determined by non-parametric bootstrapping^49^ (*N* = 10^5^) assuming the individual solvent contributions to vary linearly.

The solvation free energy for caffeine in aqueous salt solutions with varied salt concentrations are illustrated in Fig. 2A–C (for OPLS force field see Fig. S1, ESI†).

The linear tendency to increase or decrease ΔΔ*G*_sol_ (*i.e.*, caffeine solubility) with increasing salt concentration, is immediately observed for all the salts, with the slope being proportional to the Setschenow coefficient. The sign of the Setschenow coefficient indicates that all salts, but KI and CsI, *salt-out*, due to the positive Setschenow coefficient, while KI and CsI are only marginally *salting-in*. Ordering the anions according to their Setschenow coefficients (from smallest to largest) we recover in Fig. 2 the well-known Hofmeister series (I^−^ < Cl^−^ < F^−^). The ordering of cations follows their size (Cs^+^ < K^+^ < Na^+^). The variation of cation and anion contribution with the change of the counterion will be discussed in Section 3.2. When compared to the OPLS results, we found only minor quantitative differences. In specific, the Setschenow constant of KI and CsI salts changed to small positive value, *i.e.*, marginal salting-out effect is observed.

The solvation free energy of a solute can be related to its partial pressure in a liquid–vapour equilibrium^50,51^4
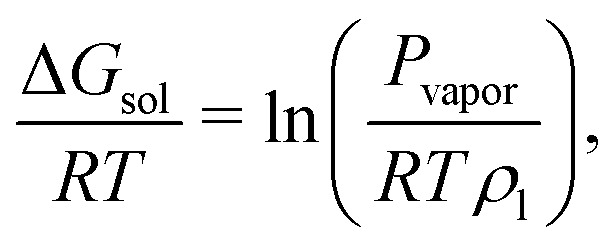
where *ρ*_l_ is the molar density in the liquid phase. In eqn (4), the vapour is assumed to be ideal and the non-ideal may be taken into account by using the fugacity in place of the vapour pressure *P*_vapor_. As described in the literature by D. Ben-Amotz *et al.*^52^ and T. R. Rogers *et al.*,^53^ Δ*G*_sol_ is related to the solvation in a fixed position. The major source of discrepancy between experimental and computational values for such small molecules as caffeine is the quality of force field. From Fig. 2A–C the solvation free energy of the caffeine monomer in pure water was found to be −16.10 ± 0.03 kcal mol^−1^ (−11.58 ± 0.02 for the OPLS force field), while the experimental value has previously been determined to −12.64 ± 0.74 kcal mol^−1^ by combination of solubility and vapour pressure measurement.^54,55^ It has been recently demonstrated by Kelly and Smith^56^ that the effect of partial charges (*i.e.*, charge derivation procedure) is decisive for the hydration free energy of caffeine, ranging from −15.2 to −11.2 kcal mol^−1^, while the value changes only marginally with the choice of the water model (∼1 kcal mol^−1^). The finite-size effects were found to be minor, up to ∼0.5 kcal mol^−1^, when increasing the number of water molecules from 500 to 8000.^56^ Moreover, a systematic decrease by 3–5 kcal mol^−1^ is observed, when on-the-fly polarisation is introduced in the free energy calculation.^56^ In this regard it is worth while mentioning that, the energy-representation theory of solvation can also be used with polarisable force fields by the introduction of an intermediate state to separately treat the many-body interactions.^57^

It is thus observed that our computed value of the solvation free energy in pure water is in the range of agreement of other calculated values with the experiment. Furthermore, the Hofmeister ordering is reproduced, allowing us to perform detailed analyses of ion-specfic effects.

### 3.2 Anion–cation contrast in caffeine solvation

To highlight the distinct roles of anion, cation, and water in the solvation of caffeine, the solvation free energy of caffeine has been decomposed into the contributions from the species present in the system. Within the framework of the energy-representation theory, we can write the decomposition as5
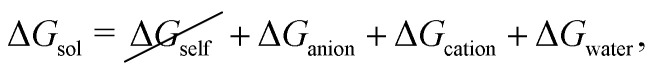
where the subscripts donate the species' contribution (expressed in more detail in eqn (7)). The self-energy correction, Δ*G*_self_, is a correction arising from the electrostatic interaction of the solute with its own images and neutralising background,^58^ and enters as a separate term in the decomposition to ensure all the contributions added up to the values presented in Fig. 2A–C. In our caffeine simulations, Δ*G*_self_ is between −0.017 and −0.015 kcal mol^−1^ (−0.041 to −0.034 kcal mol^−1^ for the simulations using the OPLS force field) over all salt concentrations and salt solutions; it can thus be neglected in the following. Similarly we can quantify the effect of the individual salt and solvent species (*i.e.*, anion, cation, and water, herein after referred to as the “solvent species”) on the Setschenow coefficient by taking the derivative of eqn (5) with respect to the salt concentration6
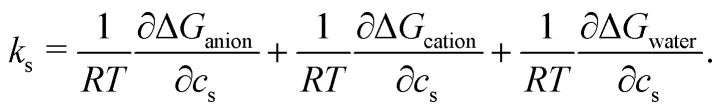
In Fig. 2D–F the decomposition of the Setschenow coefficients into the contributions arising from the individual solvent species have been visualised. The contributions from anion, cation, and water correspond to the first, second, and third terms of eqn (6), respectively. It is seen that the anions, F^−^, Cl^−^, and I^−^, are increasing the solvation free energy of caffeine, Δ*G*_sol_, with increasing salt concentration, while the cations, Na^+^, K^+^, and Cs^+^, are decreasing Δ*G*_sol_. This effectively means that the anions are *salting-out* caffeine, while cations are *salting-in*, revealing the contrast between anions and cations in altering the solubility of caffeine.

**Fig. 3:**
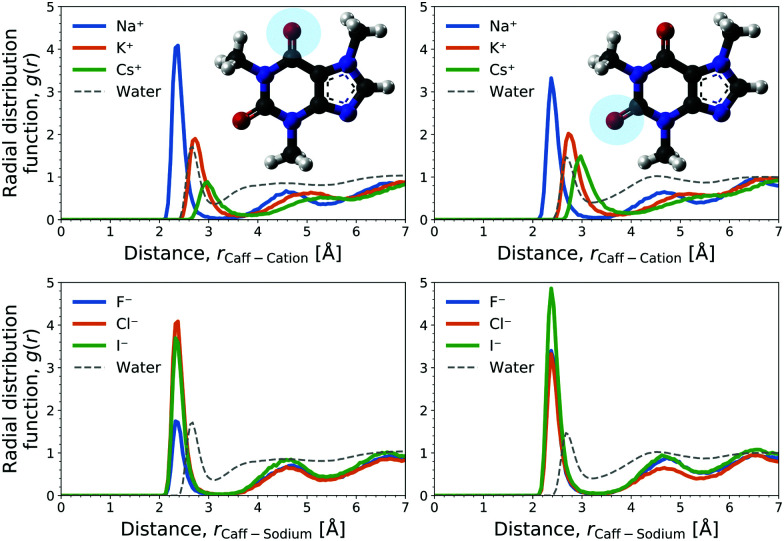
Radial distribution functions (RDF) at 1 M salt concentration of the marked oxygens of the two ketone groups of caffeine (left and right plot) with the cation for chloride salts (top) and with sodium over varied anions (bottom). The distribution of water oxygen, *i.e.*, ketone hydration, is shown for the reference.

The cation contribution is provided by the second term of eqn (6), and is more favourable (more negative) in the order of Na^+^ > K^+^ > Cs^+^. This ordering is in agreement with the Hofmeister series, but also with the ionic radii of the alkali cations. The role of the cation has been attributed to the binding to polar groups, as it is visible from radial distribution functions in Fig. 3 (see Fig. S2, ESI† for RDFs obtained with the OPLS force field) showing the enrichment of cations with respect to water. To be specific, it was found that the cations associate with the polar ketone groups of caffeine, with the height of the first peak ordered: Na^+^ > K^+^ > Cs^+^. This observation follows the same trend as the cation contribution (second term of eqn (6)) that is negative and acts to lower Δ*G*_sol_ in Fig. 2D–F. Smaller cations interact with caffeine more strongly *via* electrostatic interactions due to the possibilities to approach caffeine closer. The variation of the anion species affects the cation contribution to Δ*G*_sol_ most strongly for the sodium salts (Fig. 2D), while its effects are less yet still clearly observable for the potassium and caesium salts (Fig. 2E and F). According to the lower panel of Fig. 3, the interaction of sodium with caffeine tends to be stronger when the counter-anion is larger. This shows that the cation–caffeine interactions can be controlled by the choice of anion, with the role played by the anion size. More specifically, the order follows the anion affinity to the methyl groups (*cf.* Fig. S11, ESI†). In the vicinity of caffeine I^−^ is enriched and F^−^ is depleted. Consequently, the cation contribution to the Setschenow coefficient (Fig. 2D–F) is the strongest (most negative) when accompanying I^−^, and the weakest with F^−^. This effect is valid for every cation, and follows from local electroneutrality condition, where the increase or decrease in the anion concentration induces qualitatively same effect in the cation concentration.^35^

The anion contribution (first term of eqn (6)) is more unfavourable (more positive) in the order of F^−^ > Cl^−^ > I^−^. The order is also in agreement with the Hofmeister series for anions. The effect of accompanying cation on the anion contribution is only marginal. This stems from the fact that smaller Na^+^ is enriched more but only at close distance, compared to largest Cs^+^ cation, which is enriched less at further distances (*cf.*Fig. 3). This leads to a similar concentration of different cations in the vicinity of caffeine. In Section 3.3 we see that even when the direct interaction between the anion and caffeine is favourable, the anion contribution to the Setschenow coefficient is unfavourable due to the indirect anion-reorganisation effect (see eqn (18)). Further, in Section 3.4 we note the role of cavity formation.

According to Fig. 2D–F, the cation contributions act to increase the caffeine solubility with their negative signs and the anion and water contributions contribute oppositely. When the cation species is fixed, furthermore, the increase of the anion contribution is accompanied by the decrease of the water contribution. The water contribution to the Setschenow coefficient is positive. Still, as seen in Fig. S3 and S4, ESI,† water constitutes the largest contribution to the solvation free energy of caffeine and ensures the favourable solvation even at 2 M. Accordingly, the positive contribution from water in Fig. 2 means that the favourable contribution from water is reduced with addition of the salts and that the extent of reduction is F^−^ < Cl^−^ < I^−^ for all the cations examined. When the OPLS model is employed (Fig. S1D–F, ESI†), the orderings of the separated contributions from anion, cation, and water are less systematic with respect to the ion sizes. The anion–cation contrast still holds with OPLS, as common observations to the results in Fig. 2 from the optimised force fields for ions.

Up to this point we have been able to decompose the solvation free energy of caffeine into the contributions from anions, cations, and water, and have seen the salting-in effects of cations. The next task is to elucidate the interaction component that is responsible for the effects of salts to modulate the caffeine solubility.

### 3.3 Roles of the solute–solvent attractions and solvent reorganization

To gain insight into the mechanism of action in the modulation of the solubility of caffeine due to the individual solvent species in solution, we can examine the pairwise-energetics in the system. Within the framework of energy-representation theory of solvation, the contribution to the solvation free energy from a single solvent species (*cf.*eqn (5)) can be written as7
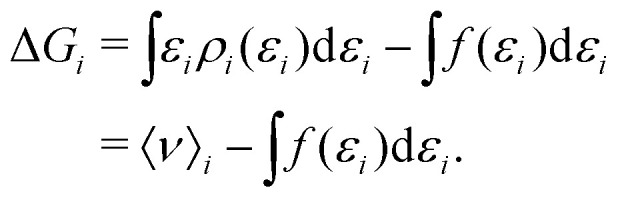
Here *ε*_*i*_ is the pair interaction energy between the solute and the solvent species *i*, 〈*ν*〉_*i*_, is the average solute–solvent interaction energy in the solution system, *ρ*_*i*_(*ε*_*i*_), is the averaged pair interaction energy distribution from the sampled configurations in the solution system, and *f*(*ε*) is a function (equal to the last two terms of eqn (18)) which takes into account the effect of solvent reorganisation. Using eqn (7) an interaction-component analysis can be conducted, by choosing the integration limits corresponding to the characteristic interaction component. The corresponding Setschenow coefficient can then be written as8
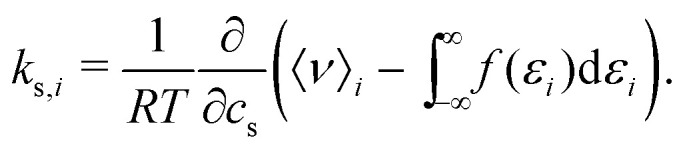
Eqn (8) involves an integration over the pair-energy value *ε*_*i*_ between solute and solvent. When *ε*_*i*_ corresponds to the hydrogen bonding, for example, *f*(*ε*_*i*_) describes the free-energy penalty due to the rearrangements of solvent structures caused by the solute–solvent hydrogen bond. When *ε*_*i*_ is much larger than *RT*, on the other hand, *f*(*ε*_*i*_) refers to the excluded-volume component, which we will discuss in the next subsection. The excluded volume is introduced by *ε*_*i*_ > *ε*_max_. The region of *ε*_*i*_ < *ε*_max_ captures such interactions as hydrogen bonding and dispersion interactions and the associated solvent-reorganisation effects, and the partial contribution from that region to the Setschenow coefficient from solvent species *i* (*i* = anion, cation, or water) is given by9
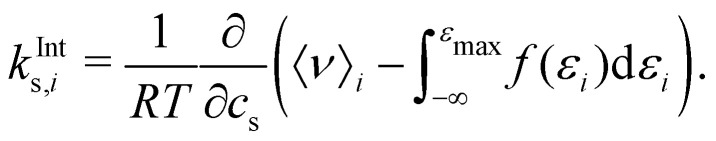
Scanning over all salts and concentrations we found *ε*_max_ = 20 kcal mol^−1^ to be an appropriate choice for both force fields. This is since *ρ*_*i*_(*ε*_*i*_) vanishes in *ε*_*i*_ > *ε*_max_ in the solution system, while the discussion in this and next subsections is not altered with any (reasonable) choice of *ε*_max_.

**Fig. 4:**
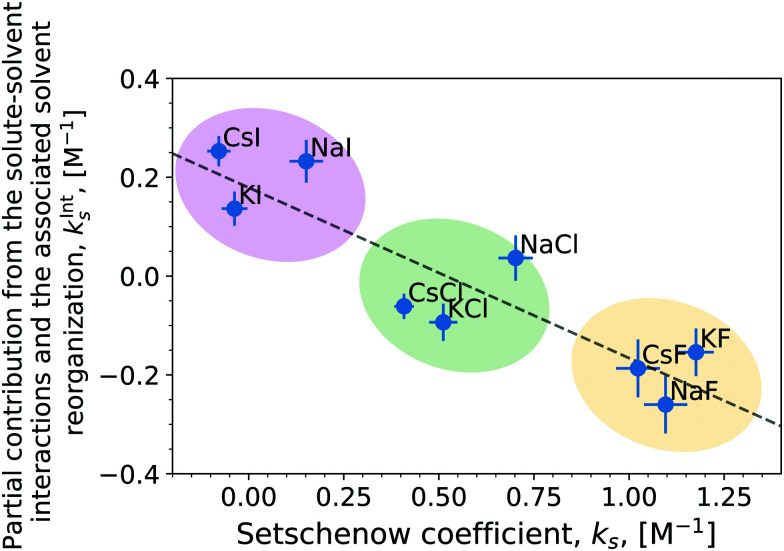
Correlation plot of the (total) Setschenow coefficient against the partial contributions from the solute–solvent interactions and the associated solvent reorganisations. The linear correlation statistics; *R*^2^: 0.809, two-sided *p*-value: 9.69 × 10^−4^.

The correlation of the total Setschenow coefficient against the partial contributions from the solute–solvent interactions and the associated solvent reorganisations has been visualised in Fig. 4 (Fig. S5, ESI† for OPLS simulations). *k*^Int^_s_ means the sum of *k*^Int^_s,*i*_ over *i* = anion, cation, and water. Evidently, the total Setschenow coefficient anti-correlates with the sum of the partial contributions *k*^Int^_s,*i*_ from anion, cation, and water. In another words, the iodide salts, which have the most negative Setschenow coefficients, exhibit positive values of *k*^Int^_s_. In contrast, the fluoride salts, which are at the opposite end of the Hofmeister series of anions and *salt-out* caffeine, possess negative *k*^Int^_s_.

Although the (total) Setschenow coefficient is well fitted linearly to the partial contributions in the correlation plot of Fig. 4, the linear fit cannot describe the cation effects on the Setschenow coefficient at fixed anions. This is evident from the feature of the correlation plot's tendency for the points to cluster around the anion rather than the cation of the various salts. This might seem slightly counter-intuitive, in light of our previous finding that the cation contribution is predominantly associated with the binding to the ketone groups of caffeine causing increased solvation of caffeine. This apparent issue is resolved by decomposing the contributions from anion, cation, and water in the regions of *ε*_*i*_ < *ε*_max_ (Fig. S6 and S7, ESI†) follow the same trends as the total Setschenow efficient in Fig. 2. The anion and water together constitute a positive contribution essentially cancelling the negative contribution by the cation. The sum of the anion, cation, and water contributions, however, follows the opposite trend, leading to the anti-correlation found in Fig. 4.

**Fig. 5:**
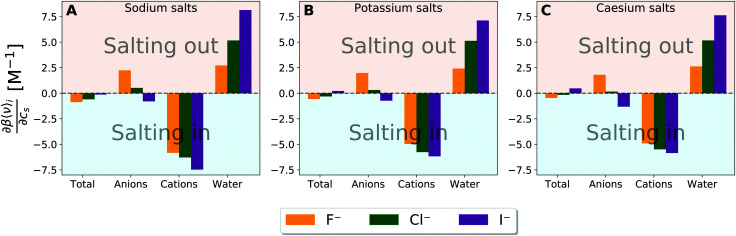
Derivative of the averaged solute–solvent interaction energy with respect to the salt concentration and the decomposed contributions originating from the solvent species namely anions, cations, and water for (A) sodium salt solutions, (B) potassiu salt solutions, and (C) caesium salt solutions. *β* is equal to the inverse of *RT*.

Up to this point, the contributions involve both the direct solute–solvent interaction and the solvent-reorganisation term. By focusing on the former, we find the clustering of points around the anion is less pronounced in the correlation between the Setschenow coefficient and the variation of solute–solvent interaction energy with increasing salt concentration (Fig. S8, ESI†). This is in strong contrast to the results obtained from the OPLS simulations, which are nearly unchanged in the salts to cluster (Fig. S9, ESI†). Fig. 5 (and Fig. S10, ESI† for the OPLS simulations) shows the derivative of the averaged solute–solvent interaction energy with respect to the salt concentration and the contributions from the anion, cation, and water. Among the decomposed contributions from the anion, cation, and water, the sign is different between Fig. 2 and 5 only for the anion contribution from iodide and additionally chloride from the OPLS simulations. This observation is most likely related to the binding of iodide anion to the methyl groups in caffeine, providing increased solvation of caffeine by protecting the hydrophobic groups. When comparing radial distribution functions between the methyl groups of caffeine and water to those with anions the anion-specificity becomes apparent (*cf.* Fig. S11 and S12, ESI†). These figures reveal a weak association of the iodide anion to caffeine, while the fluoride ions and to lesser extent the chloride ions are mainly excluded. For the OPLS force field, the same tendency is found with the exception of the chloride attraction to the methyl groups, but to lesser extent than iodide, leading to the negative anion contribution from Fig. S10 (ESI†). Actually, anions with high degrees of polarisability tend to be associated to non-polar surfaces of solutes, and this tendency is considered to bring the anion-specific effects to increase the solubilities and decrease the aggregation propensities of the solutes. However, despite the favourable interactions between caffeine and iodide, the contribution is being mainly cancelled by the contribution exerted by the water, thus causing the total effect of interactions to be more favourable by addition of fluoride and chloride salts in Fig. 5. It is speculated that the opposing driving force in the solvation of caffeine, compared to the binding of iodide to caffeine, is the decreased hydration (water contribution) of caffeine. The role of anion will be further discussed in connection to the modulation of water structure in the upcoming section with respect to the excluded-volume effect.

We now discuss the connections to experimental insights. The decomposition into the contributions from anions and cations is impossible within thermodynamics, however, efforts have been made to understand the driving forces of caffeine solvation in salt solutions on a molecular level. Monitoring ^13^C and ^1^H chemical shifts and their perturbation by sodium salts it was found that salt of weakly hydrated anions; such as NaI, NaSCN, and NaClO_4_ increased the ^13^C chemical shift.^17^ In contrast, strongly hydrated anionic salts such as NaF and NaH_2_PO_4_ yielded decreasing ^13^C chemical shifts for non-hydrogen bound carbon with increasing salt concentration. The increasing chemical shift, due to the addition of weakly hydrated anionic sodium salts, were found to fit a Langmuir binding model, providing an interpretation to the change in chemical shifts and allowing the estimation of the binding free energies. The NMR observations are consistent with previous finding by experiments and simulations, that weakly hydrated anions can bind to non-polar surface patches of proteins.^59–63^ However, from the simulations presented here, we observe *both* the association of weakly hydrated anions like iodide to the hydrophobic methyl groups of caffeine and the association of cations to the polar ketone groups as revealed from the radial distribution functions (compare Fig. 3 with Fig. S2 and compare Fig. S11 with Fig. S12, ESI†). Actually, the anion effect is often analysed by fixing the cation to sodium and the role of anion–cation interaction has not been explored in depth. The association of cations to negatively charged groups is also observed on protein surfaces. The cations prefer to bind to negatively charged amino-acid residues, *i.e.*, aspartate and glutamate, and to a lesser extent also to amide moieties, with smaller sodium cations binding tighter than potassium.^35,64–66^ The experimental chemical shifts could reflect both the binding of anions to the methyl groups and the binding of sodium cation to the ketone groups of caffeine.

### 3.4 Excluded-volume effects can be attributed to the anion as revealed by interaction-component analysis

It has previously been hypothesised that the *salting-out* of caffeine by sodium salts is due to excluded-volume effects by the associated anion.^16,67^ The effect of excluded volume can be addressed systematically on the basis of eqn (7) and (8). The excluded-volume contribution of solvent species *i* (*i* is anion, cation, or water) to the Setschenow coefficient *k*^EV^_s,*i*_ is provided by10
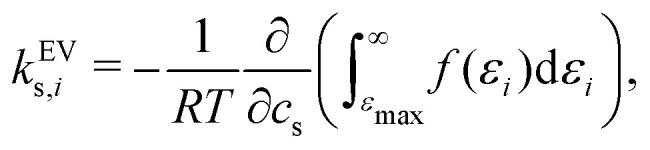
where *ε*_max_ is the same as the one in eqn (9). In eqn (10), the contribution from the high-energy region with solute–solvent overlap is quantified.

**Fig. 6:**
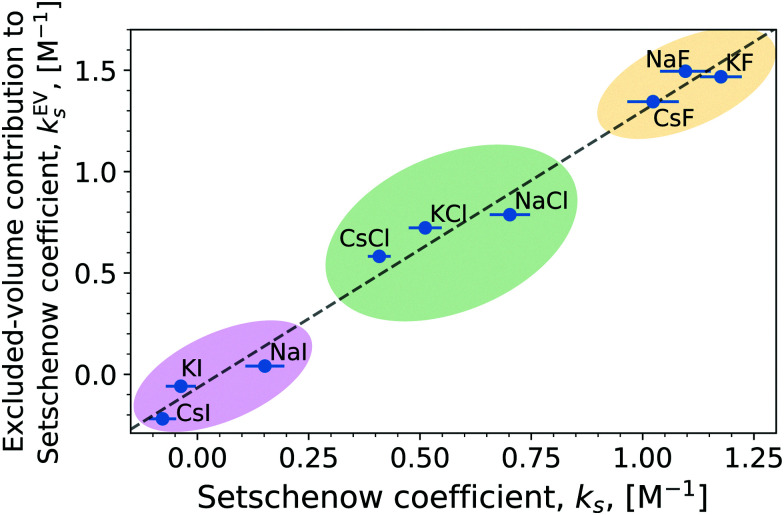
Linear correlation between the total Setschenow coefficient and the excluded-volume contribution to the Setschenow coefficient obtained from integration over the high-energy region of the energy coordinate. The linear correlation statistics; *R*^2^: 0.978, two-sided *p*-value: 4.77 × 10^−7^.

Fig. 6 (and Fig. S13, ESI† for the OPLS simulations) shows the correlation between the Setschenow coefficient and the net excluded-volume contribution given by the sum of *k*^EV^_s,*i*_ over *i* = anion, cation, and water. A linear correlation indeed exists (*R*^2^: 0.978, two-sided *p*-value: 4.77 × 10^−7^) between them. It is also noteworthy that the salts tend to cluster around anion, with F^−^ > Cl^−^ > I^−^ for both the Setschenow coefficient and the excluded-volume contribution. The dependence of the Setschenow coefficient on the ionic species reflects mainly the anion dependence of the excluded-volume effect.

**Fig. 7:**
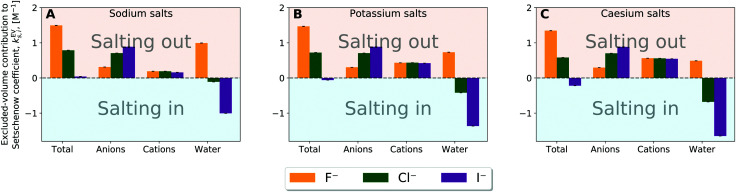
Species decomposition of the excluded-volume component into the contributions from anion, cation, and water for (A) sodium salt solutions, (B) potassium salt solutions, and (C) caesium salt solutions. Error bars report the standard error determined by non-parametric bootstrapping^49^ (*N* = 10^5^) assuming that the individual solvent contributions vary linearly.

To see the role of each of anion, cation, and water in the excluded-volume effect, Fig. 7 (and Fig. S14, ESI† for the simulations using the OPLS force field) shows *k*^EV^_s_ for the individual solvent species. *k*^EV^_s_ is positive for all the anions and cations. The excluded-volume effect is related to the energetics required to create a cavity for the solvation. The excluded-volume contribution from an anion or cation thus emerges with addition of salts, leading to a positive *k*^EV^_s_. It can be expected furthermore that *k*^EV^_s_ is correlated to the ion size since the excluded-volume contribution to the solvation free energy is the free-energy penalty to displace solvent molecules. The correlation to the ion size exists indeed (Fig. S15 and S16, ESI†), with the excluded-volume contribution from the anion or cation linearly correlating with the ionic radius, which are modelled as the parameter *σ* in the Lennard-Jones potential. The correlation is, however, not as great for the OPLS simulations. In case of the OPLS force-field, not only the *σ*, but also the *ε* value is varied between the anions. Consequently, the *σ* parameter *alone* is not a quantitative descriptor of the ion size and neither of the excluded volume.^68^

The excluded-volume effect of water can act to *salt-out* for the fluoride salts and to *salt-in* for the chloride and iodide salts. This observation is related to the perturbation in water structure by the chaotropic/kosmotropic properties of the ions. Iodide and chloride are known as “water-structure breakers” (chaotropic) thus making it energetically less costly and even favourable to create the cavity required to introduce the solute into the system. On the other hand the fluoride anion is known as a “water-structure maker” (kosmotropic) thus making the cavity formation more energetically costly. In general, the excluded-volume component from water is larger (more positive) when particle density of water is higher. Fig. S17 and S18 (ESI†) then provide correlation plots between the change in the water particle density upon addition of salt and the water contribution to the excluded-volume component in the Setchenow coefficient. A strong correlation is observed. When the particle density of water reduces upon addition of a salt, the water contribution to the excluded-volume component is correspondingly smaller. The chaotropic and kosmotropic properties of ions thus describe well the ordering of the excluded-volume effect of water on the solubility.

## 4 Concluding remarks

The roles of anion and cation are different in the perturbation on solvation. In this study we highlight these differences caused by the ion size and sign of charge. The major perturbation of salt has been found to be (a) the strengthening or weakening of water structure in connection to the formation of the cavity required to introduce a caffeine molecule, and (b) the binding or exclusion of salt ions and water. They are strongly connected to the strength of anion–cation binding and the change in the water particle density upon addition of salt.

The properties and underlying physics of water has been investigated exhaustively over time, however, with new discoveries still being made today.^69^ For example we know that there is an intrinsic offset in the size between cations and anions to achieve the same difference in entropy change of water upon addition of salt. Fluoride and lithium have nearly the same effects of disordering water, however, the ionic size is much larger for the former.^70,71^ This observation has been attributed to the idea of anions interacting more strongly with water than cations for a given ion radius as a consequence of the asymmetry of water's dipole. The difference in hydration of ions of the same sign, but different size is credited to the balance between the ordering of water around the ion by electrostatic interactions and the water–water ordering to achieve hydrogen bonding as it would be characteristic for a hydrophobic solute.^69^

Through the decomposition of the free energy of solvation of caffeine by various species constituting the solution, we extracted the effects of solute interactions with individual species in the solution and that of the excluded volume. While we find, in agreement with conventional idea,^16,17,24,72,73^ that the solvation of caffeine in salt solutions is highly anion-dependent, this is attributed to the excluded-volume effect, rather than the change in the direct interactions of caffeine. To provide stronger evidence of this, a future study of even more weakly hydrated anionic salts than iodide salts, such as thiocyanate and perchlorate salts would be advantageous.

Combining molecular simulation and energy-representation theory of solvation has proven useful. In particular, the ability to disentangle the net effect to the role of the individual solvent species on the solute stability can be insightful to understand and engineer the solvation effects that appear in a variety of fields. Within protein chemistry the effects of solvents and co-solvents are still of great interest as protein (mis)folding^74–76^ and aggregation^77–80^ are strongly connected to the protein's interactions with the solvent. Since the method allows for the selection of solvent and solute arbitrarily, it is possible to define a selection of amino-acid residues as the solute, while the remaining part of the protein and water can be considered the solvent. This enables the possibility to study the modulation of enzymatic activity due to changes in amino-acid residues of the active sites of enzymes and in the solvent compositions.^81^ Another application is the possibility to study the strengthening of stabilising interactions between pairs of residues favouring specific folded states of proteins,^82,83^ to withstand extreme environments found in industrial settings.

## Conflicts of interest

There are no conflicts to declare.

## Supplementary Material

CP-024-D1CP04129K-s001

## Appendices

### Appendix A Statistical mechanical expression for the solvation free energy and the energy-representation theory

The chemical potential in the isothermal–isobaric ensemble is given as the difference in Gibbs free energy upon insertion of a solute species. The chemical potential can be written as

11

where *k*_B_ is the Boltzmann constant and Δ is the isothermal–isobaric partition function at *n* number of solute molecules, *N* number of solvent molecules, temperature *T*, and pressure *P*. *μ* is given by

12

where *λ*_t_ is the thermal wavelength for the solute, *V* is the system volume, *Γ*_*q*_ is the configurational space, and *q̄* and *q* donate collectively the coordinates of the solute and solvent particles, respectively. *U*_t_(*q̄*) is the intramolecular energy of the solute particle, *U*_o_(*q*) is the intra- and intermolecular energy among the solvent particles, and *U*_t,o_(*q̄*,*q*) is the interaction energy between the solute and solvent.

Further assuming the system is homogeneous and that the potential energy functions are pairwise additive, eqn (12) may be rewritten as

13

In eqn (13) the first term is the ideal term, the second term, *G*_iso_ is the free energy of the solute molecule in a vacuum and fixed in space, and the third term is the solvation free energy, yielding the free energy of turning on the interactions between the solute and the solvent given by

14

The solvation free energy may be calculated *via* the Kirkwood charging formula which relates the solvation free energy to pair correlation functions *via* a series of intermediate states *λ*

15

where *u*_*λ*_ is the solvent–solvent pair energy, *ρ*_*λ*_ is the solute–solvent distribution function, and *q* refers here to the relative configuration of a solvent molecule to the solute. While the Kirkwood charging formula is an exact expression, it is of difficulty for practical computation when the solvent-solvent density distribution function is represented over a high-dimensional set of coordinates *q*. However, utilising a coordinate of reduced dimension one may obtain useful expressions. Within the energy-representation theory, the chosen coordinate is the pair-interaction energy, in which the instantaneous pair-interaction energy distribution, , may be obtained at any given configuration by the definition

16

where the summation is taken over all the solvent particles and *q*_*i*_ is the configuration of the *i*-th solvent molecule. The Kirkwood charging formula within the energy-representation is then given by

17

where *ρ*^e^_*λ*_ is the *λ*-dependent ensemble average of eqn (16). Conducting partial integration and separating the direct and indirect parts of the potential of the mean force, we obtain

18

In eqn (18) the first term is equal to the average solute–solvent pair-interaction at full coupling (*λ* = 1), and the second term yields the pair entropy within the energy representation. The third term takes into account the effect of solvent-solvent correlations (indirect part of the PMF), and is in this work approximated by a combined Percus-Yevick (PY)-type and hypernetted-chain (HNC)-type functional, as it has been done in previous work employing a similar strategy.^28,42,84,85^
