# Supplementary material for: Anion–cation contrast of small molecule solvation in salt solutions
Source: Phys Chem Chem Phys. 2022 Jan 4;24(5):3238–49. doi: 10.1039/d1cp04129k (PMC8809138; doi:10.1039/d1cp04129k)
Supplement: CP-024-D1CP04129K-s001 [file CP-024-D1CP04129K-s001.pdf]

Electronic Supplementary Material (ESI) for Physical Chemistry Chemical Physics.
This journal is © the Owner Societies 2022

Supplementary Information: Anion-Cation Contrast of Caffeine Solvation in Salt Solutions

Stefan Hervø-Hansen,[1] Jan Heyda,[2] Mikael Lund,[3] and Nobuyuki Matubayasi[4]

Contents

1 Supplementary Figure 1: Solvation free energy of caffeine with salt and the Setschenow Coefficient. (OPLS) 3

2 Supplementary Figure 2: Radial distribution functions between the ketone groups of caffeine and cations. (OPLS) 4

3 Supplementary Figure 3: Species decomposition of solvation free energy of caffeine. 5

4 Supplementary Figure 4: Species decomposition of solvation free energy of caffeine. (OPLS) 6

5 Supplementary Figure 5: Correlation between the Setschenow coefficient and the partial contributions from the solute-solvent interactions and the associated solvent reorganisations. (OPLS) 7

6 Supplementary Figure 6: Species decomposition of the solute-solvent interactions and the associated solvent reorganisations. 8

7 Supplementary Figure 7: Species decomposition of the solute-solvent interactions and the associated solvent reorganisations. (OPLS) 8

8 Supplementary Figure 8: Correlation between the Setschenow coefficient and the derivative of the averaged solute-solvent interaction energy with respect to the salt concentration. 9

9 Supplementary Figure 9: Correlation between the Setschenow coefficient and the derivative of the averaged solute-solvent interaction energy with respect to the salt concentration. (OPLS) 10

10 Supplementary Figure 10: Species decomposition of the solute-solvent interaction energy with resepct to salt concentration. (OPLS) 11

11 Supplementary Figure 11: Radial distribution functions between the methyl groups of caffeine and anions. 12

[1]Division of Theoretical Chemistry, Department of Chemistry, Lund University, Lund SE 221 00, Sweden. Tel: +46 46 222 8251; E-mail: stefan.hervo_hansen@teokem.lu.se
[2]Department of Physical Chemistry, University of Chemistry and Technology, Prague CZ-16628, Czech Republic. Tel: +420 22044 4297; E-mail: heydaj@vscht.cz
[3]Division of Theoretical Chemistry, Department of Chemistry, Lund University, Lund SE 221 00, Sweden. Tel: +46 46 222 3167; E-mail: mikael.lund@teokem.lu.se
[4]Division of Chemical Engineering, Graduate School of Engineering Science, Osaka University, Toyonaka, Osaka 560-8531, Japan. Fax: +81-6-6850-6343; Tel: +81-6-6850-6565; E-mail: nobuyuki@cheng.es.osaka-u.ac.jp

12 Supplementary Figure 12: Radial distribution functions between the methyl groups of caffeine and anions. (OPLS) 13

13 Supplementary Figure 13: Correlation between the Setschenow coefficient and the excluded-volume component. (OPLS) 14

14 Supplementary Figure 14: Species decomposition of the excluded-volume component. (OPLS) 15

15 Supplementary Figure 15: Correlation between the excluded-volume contribution from the anion or cation and the ionic radius. 16

16 Supplementary Figure 16: Correlation between the excluded-volume contribution from the anion or cation and the ionic radius. (OPLS) 17

17 Supplementary Figure 17: Correlation between the derivative of the water molarity with respect to the salt concentration and the water contribution to the excluded-volume component in the Setschenow coefficient. 18

18 Supplementary Figure 18: Correlation between the derivative of the water molarity with respect to the salt concentration and the water contribution to the excluded-volume component in the Setschenow coefficient. (OPLS) 19

1 Supplementary Figure 1: Solvation free energy of caffeine with salt and the Setschenow Coefficient. (OPLS)

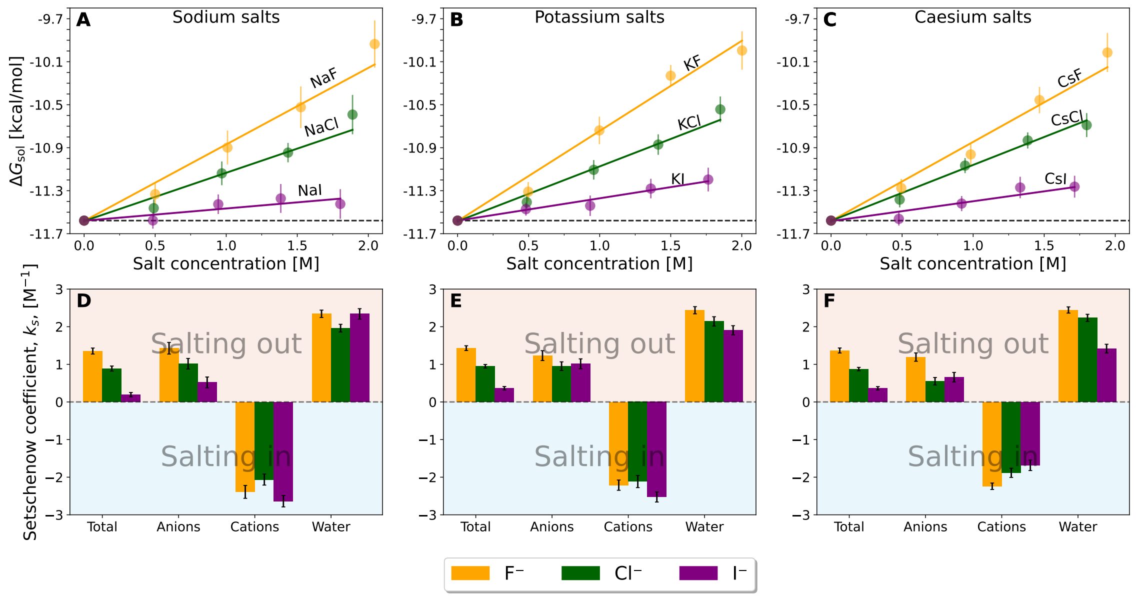

Figure S1: Solvation free energy, ΔG_{sol}, of caffeine as a function of salt concentration using the OPLS force field (*top*) **A.** sodium salts, **B.** potassium salts, and **C.** caesium salts with the colour differentiating the corresponding anion as donated by the legend. Setschenow coefficient, k_s, and the contributions from the solvent species (cf. Eq. 6) namely anions, cations, and water (*bottom*) for **D.** sodium salt solutions, **E.** potassium salt solutions, and **F.** caesium salt solutions. The anion, cation, and water contributions in D-F correspond to the first, second, and third terms of Eq. 6. The self-energy correction was found not to vary with increasing salt concentration. The error bars shown report the 95% confidence interval with the errors for the Setschenow coefficients being determined by non-parametric bootstrapping (B. Efron, *Biometrika*, 1981,68, 589–599 using 10^5 iterations and assuming the individual solvent contributions to vary linearly.

2 Supplementary Figure 2: Radial distribution functions between the ketone groups of caffeine and cations. (OPLS)

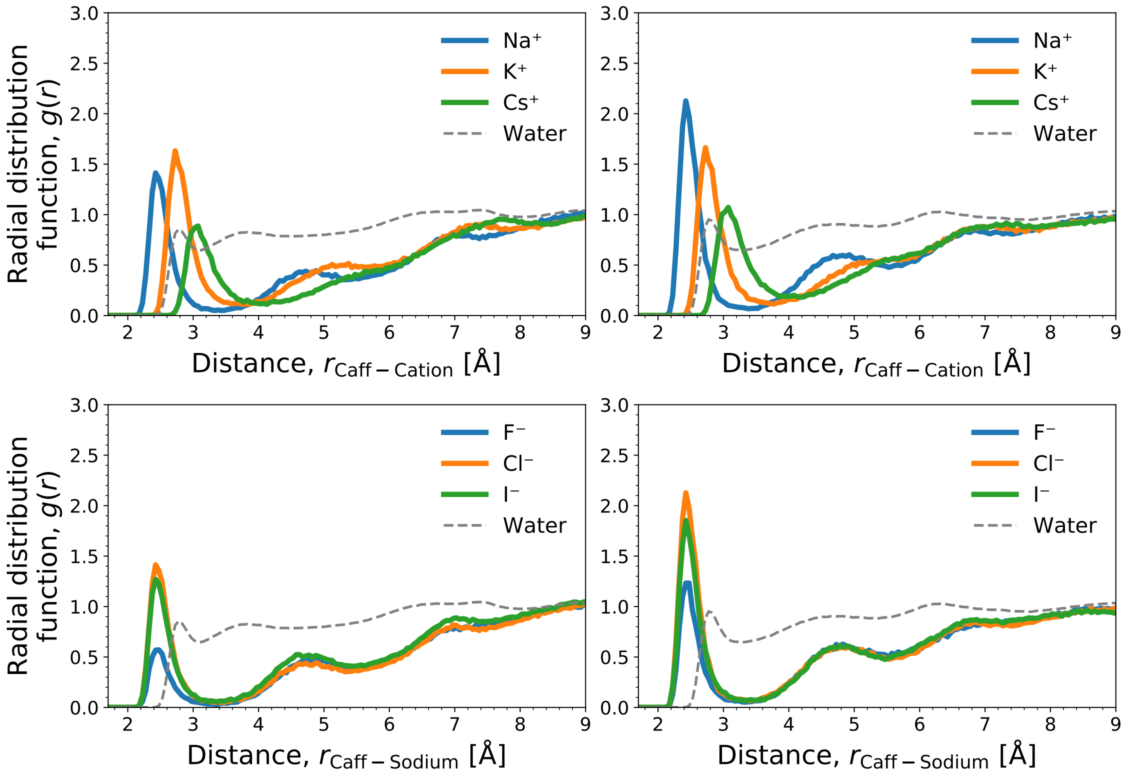

Figure S2: Radial distribution functions (RDF) at 1 M salt of the marked oxygens of the two ketone groups of caffeine (left and right plot) with the cation for chloride salts (top) and with sodium over varied anions (bottom). The distribution of water oxygen, i.e., ketone hydration, is shown for the reference. The OPLS force field was used here.

3 Supplementary Figure 3: Species decomposition of solvation free energy of caffeine.

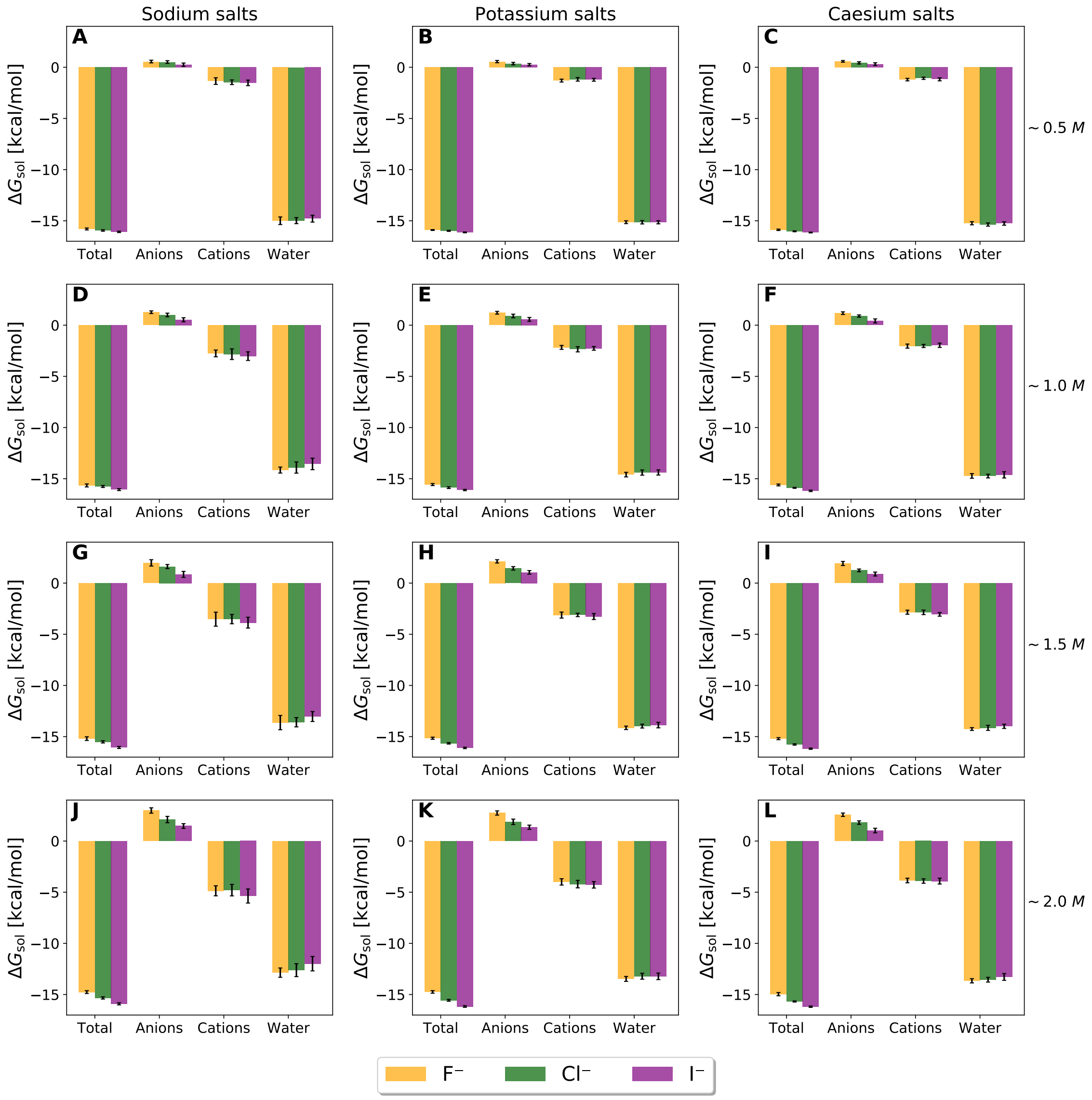

Figure S3: Solvation free energy, ΔG_{sol} of caffeine and the contributions exerted by the individual solvent species. The error bars shown report the 95% confidence interval averaging over the 25 individual simulations (each of 50 ns length). The concentrations written on the right are approximate, and their precise values appear in the abscissa of Fig. 2**A-C**. The addition of the individual solvent species contributions is different from the total by the self energy ΔG_{self}. See the discussion concerning Eq. 5.

4 Supplementary Figure 4: Species decomposition of solvation free energy of caffeine. (OPLS)

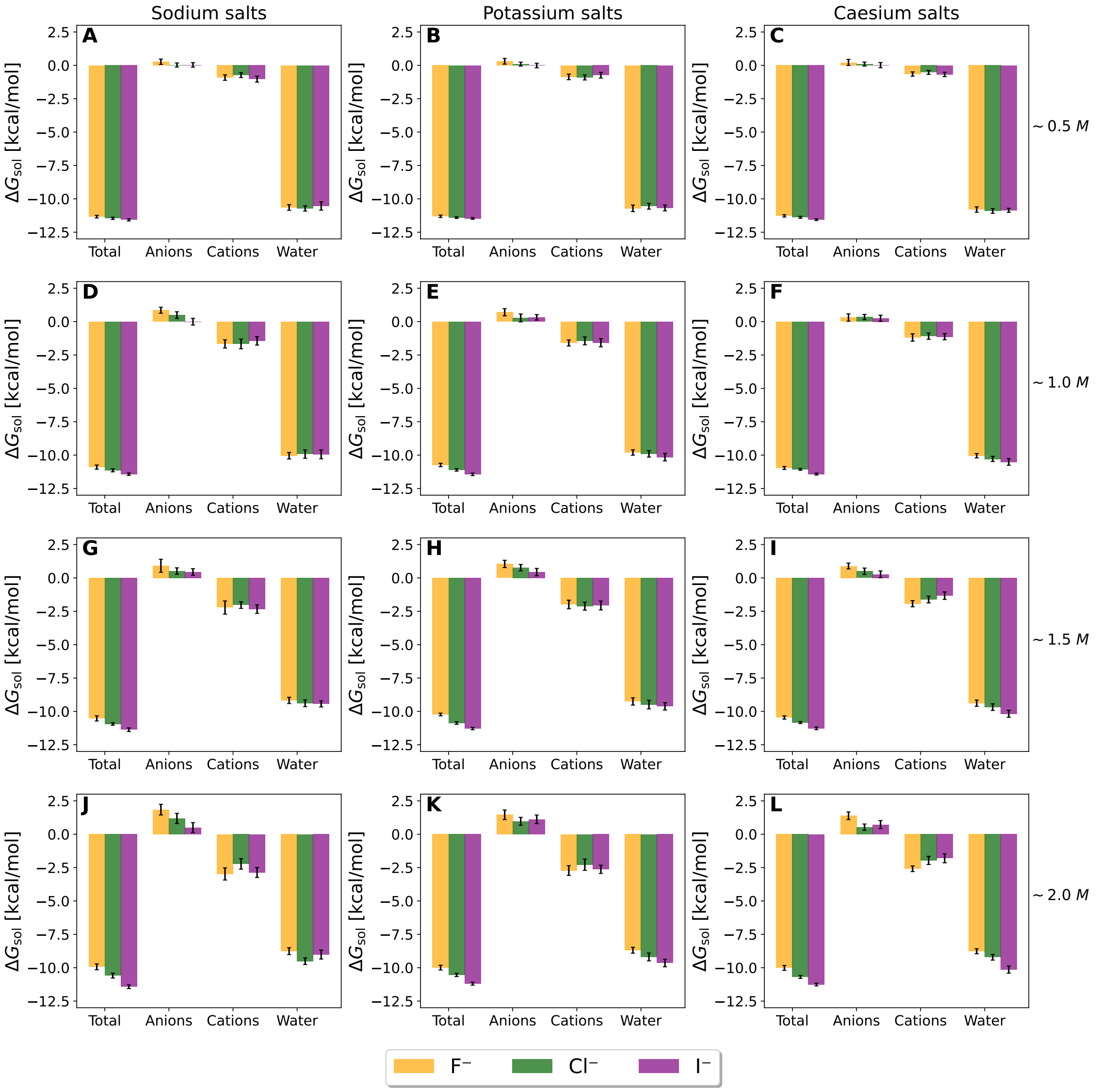

Figure S4: Solvation free energy, ΔG_{sol} of caffeine and the contributions exerted by the individual solvent species using the OPLS force field. The error bars shown report the 95% confidence interval averaging over the 25 individual simulations (each of 50 ns length). The concentrations written on the right are approximate, and their precise values appear in the abscissa of Fig. S1**A-C**. The addition of the individual solvent species contributions is different from the total by the self energy ΔG_{self}. See the discussion concerning Eq. 5.

5 Supplementary Figure 5: Correlation between the Setschenow coefficient and the partial contributions from the solute-solvent interactions and the associated solvent reorganisations. (OPLS)

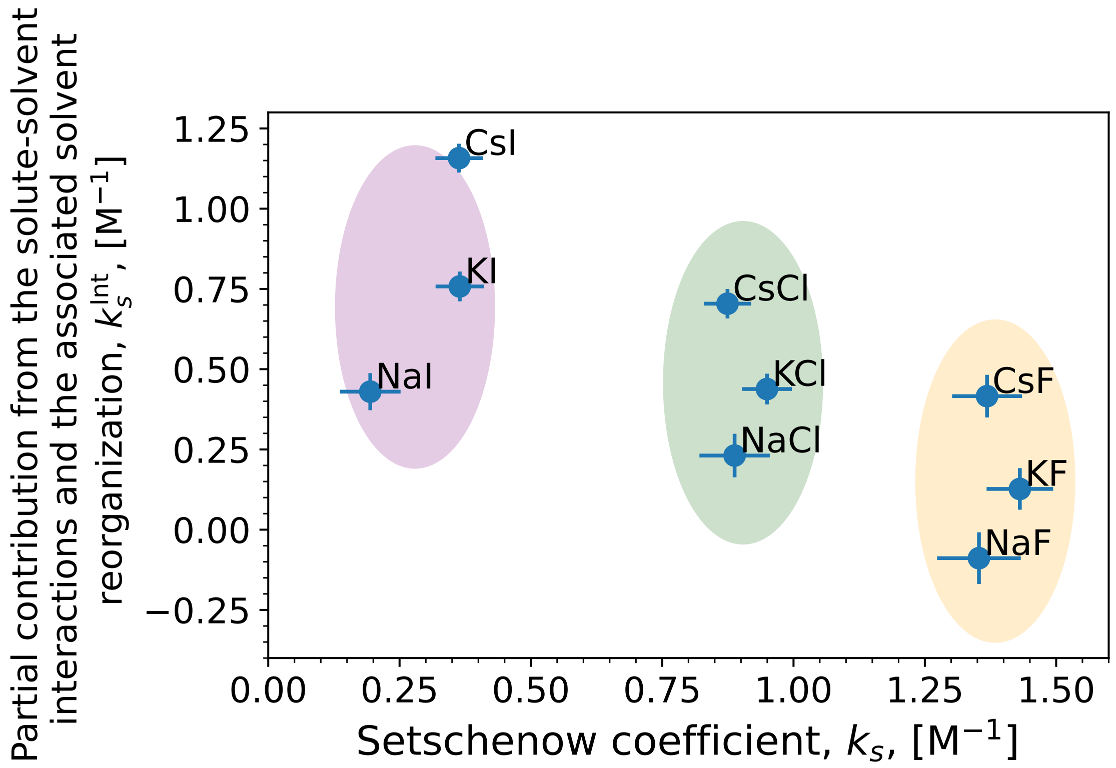

Figure S5: Correlation plot of the (total) Setschenow coefficient against the partial contributions from the solute-solvent interactions and the associated solvent reorganisations. The linear correlation statistics; R^2: 0.475, two-sided p-value: 0.0401.

6 Supplementary Figure 6: Species decomposition of the solute-solvent interactions and the associated solvent reorganisations.

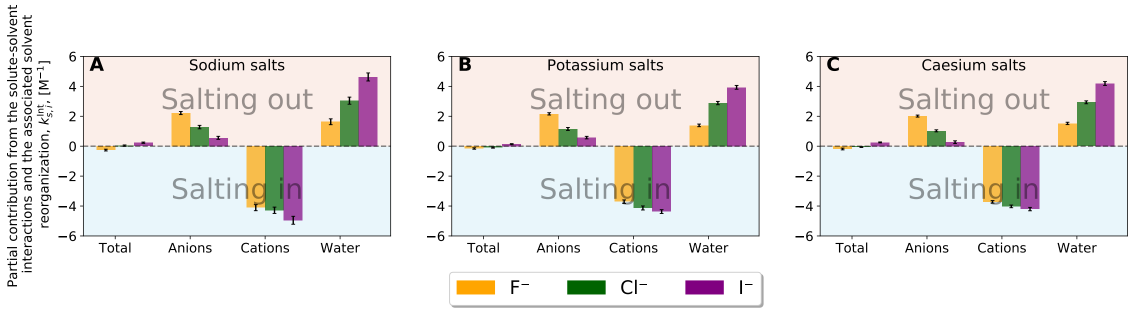

Figure S6: Species decomposition of the solute-solvent interactions and the associated solvent reorganisations for **A** sodium salts, **B** potassium salts, and **C** caesium salts. The error bars shown report the 95% confidence interval determined by non-parametric bootstrapping (B. Efron, *Biometrika*, 1981,68, 589–599) using $N = 10^5$ iterations and assuming the individual solvent contributions to vary linearly.

7 Supplementary Figure 7: Species decomposition of the solute-solvent interactions and the associated solvent reorganisations. (OPLS)

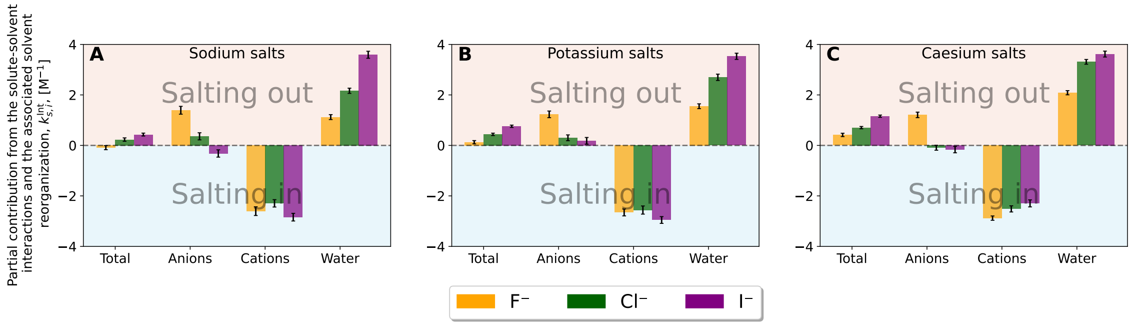

Figure S7: Species decomposition of the solute-solvent interactions and the associated solvent reorganisations for **A** sodium salts, **B** potassium salts, and **C** caesium salts. The error bars shown report the 95% confidence interval determined by non-parametric bootstrapping (B. Efron, *Biometrika*, 1981,68, 589–599) using $N = 10^5$ iterations and assuming the individual solvent contributions to vary linearly. The OPLS force field was used here.

8 Supplementary Figure 8: Correlation between the Setschenow coefficient and the derivative of the averaged solute-solvent interaction energy with respect to the salt concentration.

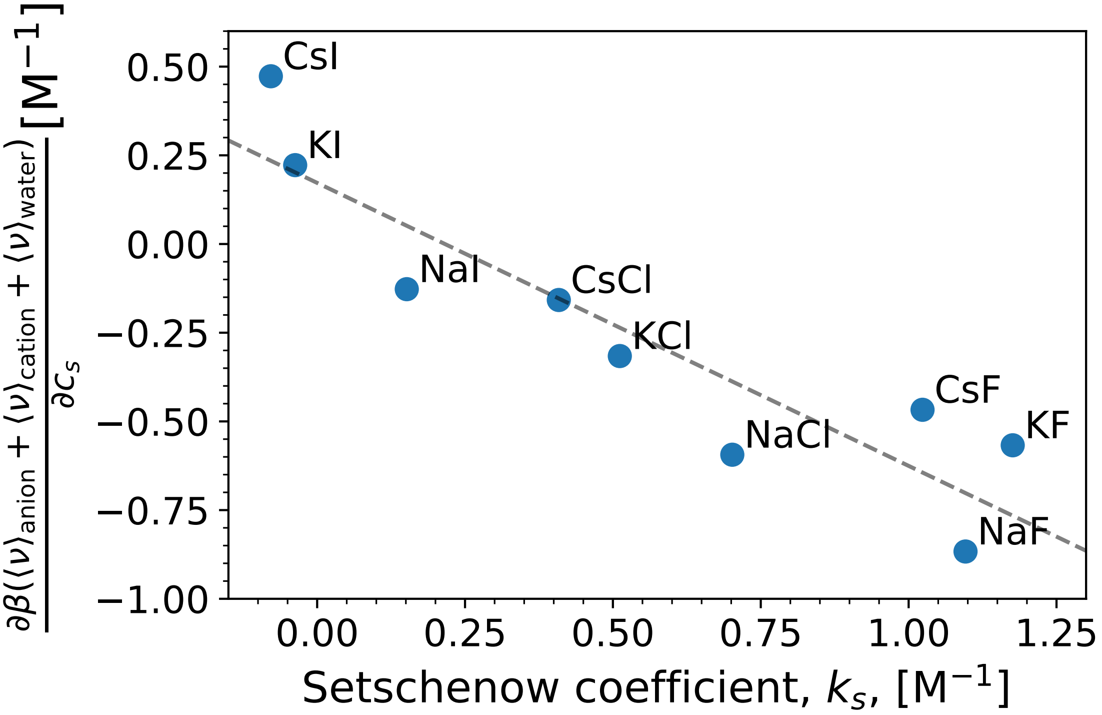

Figure S8: Correlation plot between the Setschenow coefficient and the derivative of the averaged solute-solvent interaction energy with respect to the salt concentration. The value on the y-axis is the total value in Figure 5. The linear correlation statistics; R^2: 0.834, two-sided p-value: 5.85×10^{-4}.

9 Supplementary Figure 9: Correlation between the Setschenow coefficient and the derivative of the averaged solute-solvent interaction energy with respect to the salt concentration. (OPLS)

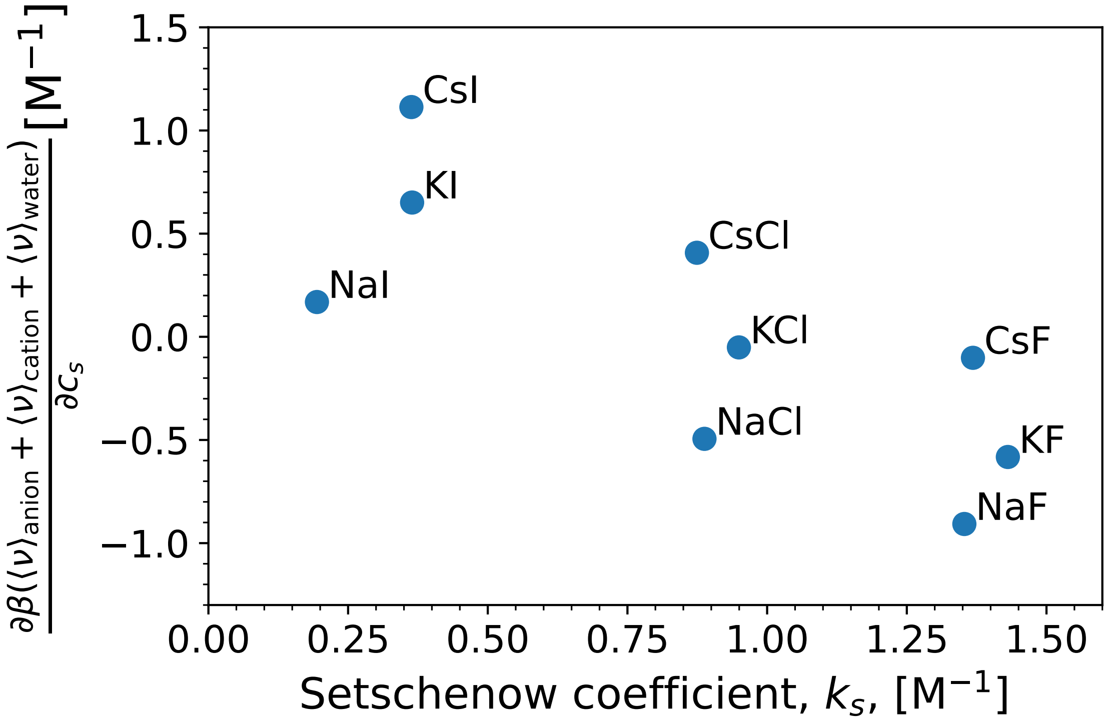

Figure S9: Correlation plot between the Setschenow coefficient and the derivative of the averaged solute-solvent interaction energy with respect to the salt concentration. The value on the y-axis is the total value in Figure S10. The linear correlation statistics; R^2: 0.579, two-sided p-value: 0.0172. The OPLS force field was used here.

10 Supplementary Figure 10: Species decomposition of the solute-solvent interaction energy with resepct to salt concentration. (OPLS)

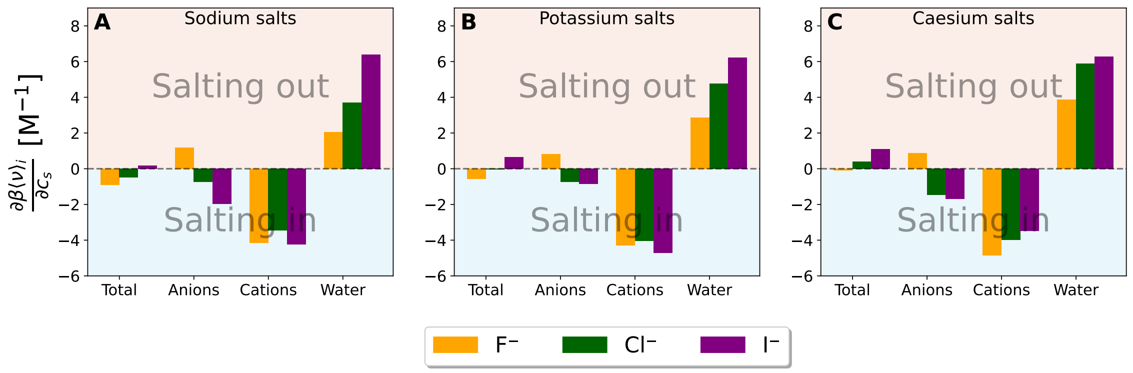

Figure S10: Derivative of the averaged solute-solvent interaction energy with respect to the salt concentration and the decomposed contributions originating from the solvent species namely anions, cations, and water for **A.** sodium salt solutions, **B.** potassium salt solutions, and **C.** caesium salt solutions. β is equal to the inverse of RT. The OPLS force field was used here.

11 Supplementary Figure 11: Radial distribution functions between the methyl groups of caffeine and anions.

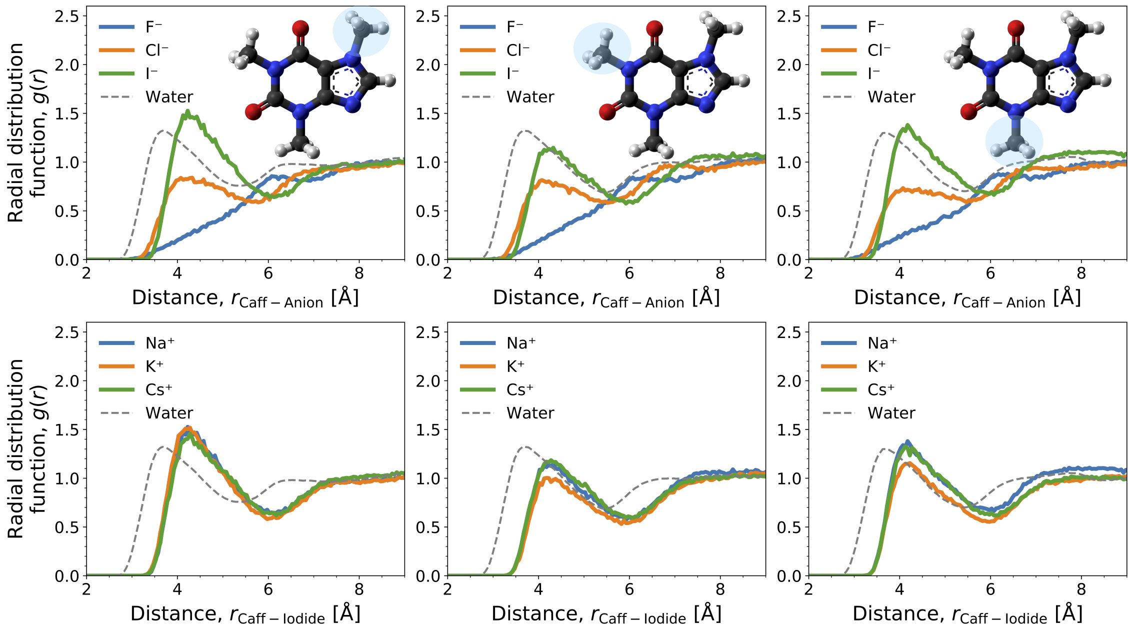

Figure S11: Radial distribution functions at 1 M of the three methyl groups of caffeine (columns) with the anions of sodium salts (top) namely F^-, Cl^-, and I^- and with I^- in NaI, KI, and CsI salts (bottom). The distribution of water oxygen, i.e., methyl hydration, is shown for the reference.

12 Supplementary Figure 12: Radial distribution functions between the methyl groups of caffeine and anions. (OPLS)

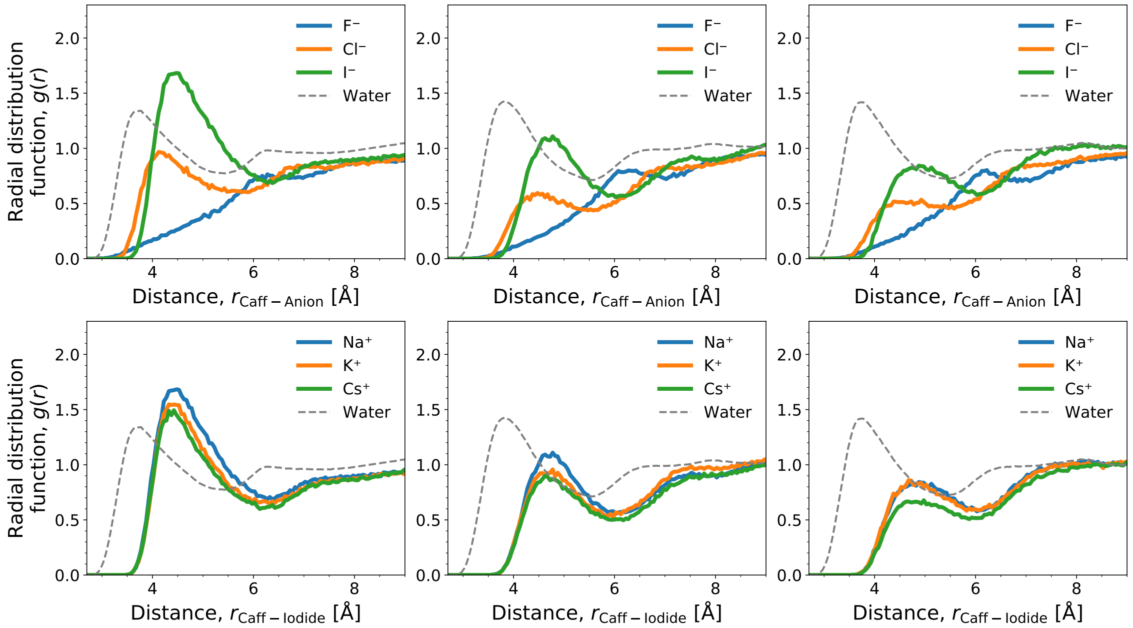

Figure S12: Radial distribution functions at 1 M salt using the OPLS force field of the three methyl groups of caffeine (columns) with the anions of sodium salts (top) namely F^-, Cl^-, and I^- and with I^- in NaI, KI, and CsI salts (bottom). The distribution of water oxygen, i.e., methyl hydration, is shown for the reference. The OPLS force field was used here.

13 Supplementary Figure 13: Correlation between the Setschenow coefficient and the excluded-volume component. (OPLS)

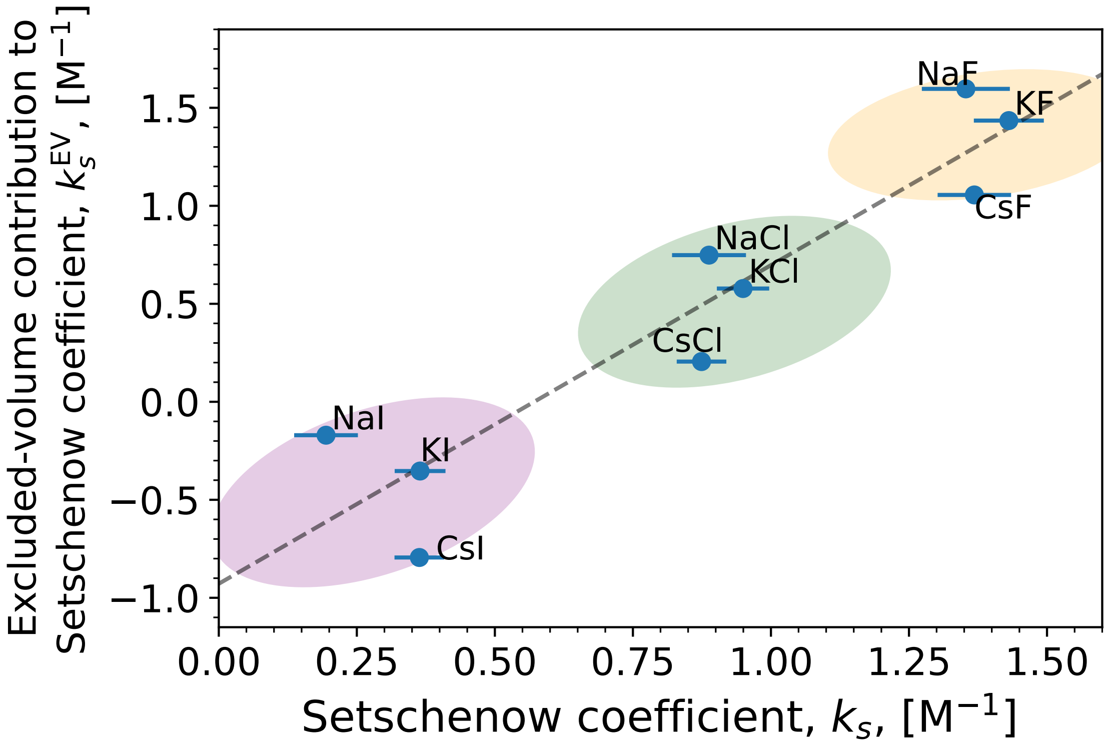

Figure S13: Linear correlation between the total Setschenow coefficient and the excluded-volume contribution to the Setschenow coefficient obtained from integration over the high-energy region of the energy coordinate. The linear correlation statistics; R^2: 0.869, two-sided p-value: 2.52×10^{-4}. The OPLS force field was used here.

14 Supplementary Figure 14: Species decomposition of the excluded-volume component. (OPLS)

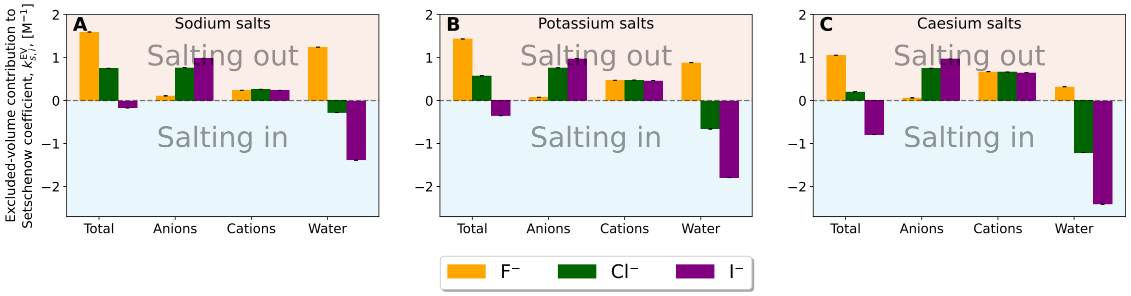

Figure S14: Species decomposition of the excluded-volume component into the contributions from anion, cation, and water for **A.** sodium salt solutions, **B.** potassium salt solutions, and **C.** caesium salt solutions. Error bars report the standard error determined by non-parametric bootstrapping(B. Efron, *Biometrika*, 1981,68, 589–599 using 10^5 iterations and assuming that the individual solvent contributions vary linearly. The OPLS force field was used here.

15 Supplementary Figure 15: Correlation between the excluded-volume contribution from the anion or cation and the ionic radius.

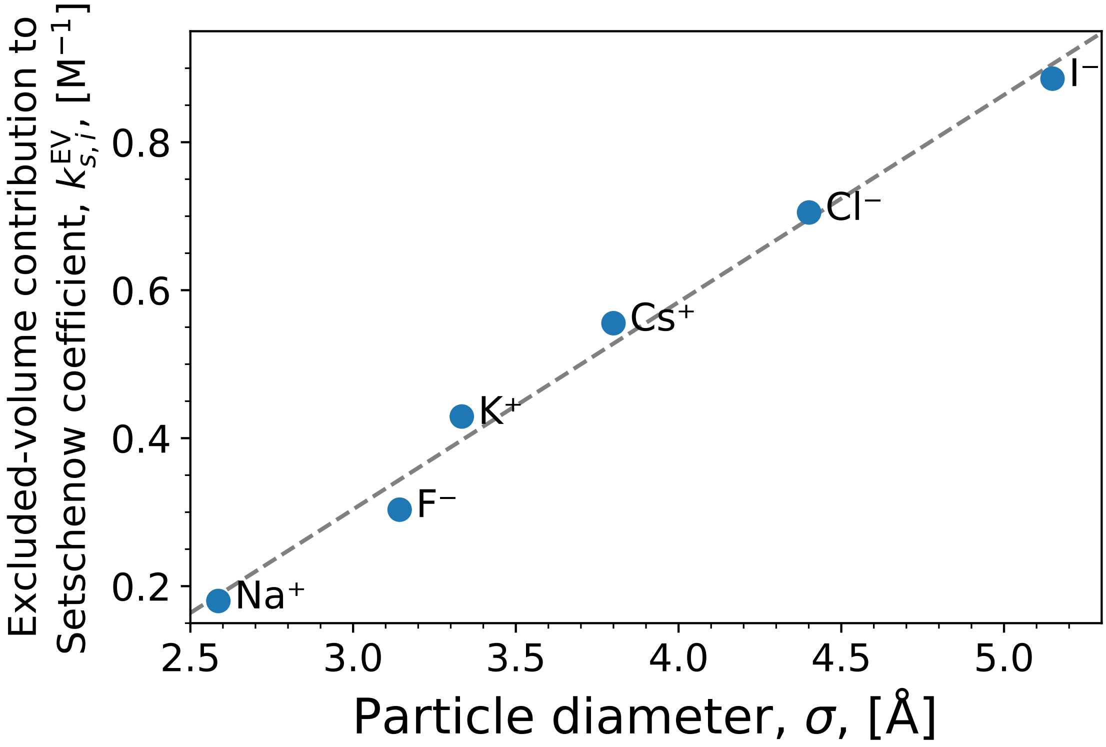

Figure S15: Excluded-volume contribution to the Setschenow coefficient as a function of the particle diameter σ from the Lennard-Jones potential. The error bars (standard deviation) are smaller than the symbol size. The linear correlation statistics; R^2: 0.988, two-sided p-value: 5.09×10^{-5}.

16 Supplementary Figure 16: Correlation between the excluded-volume contribution from the anion or cation and the ionic radius. (OPLS)

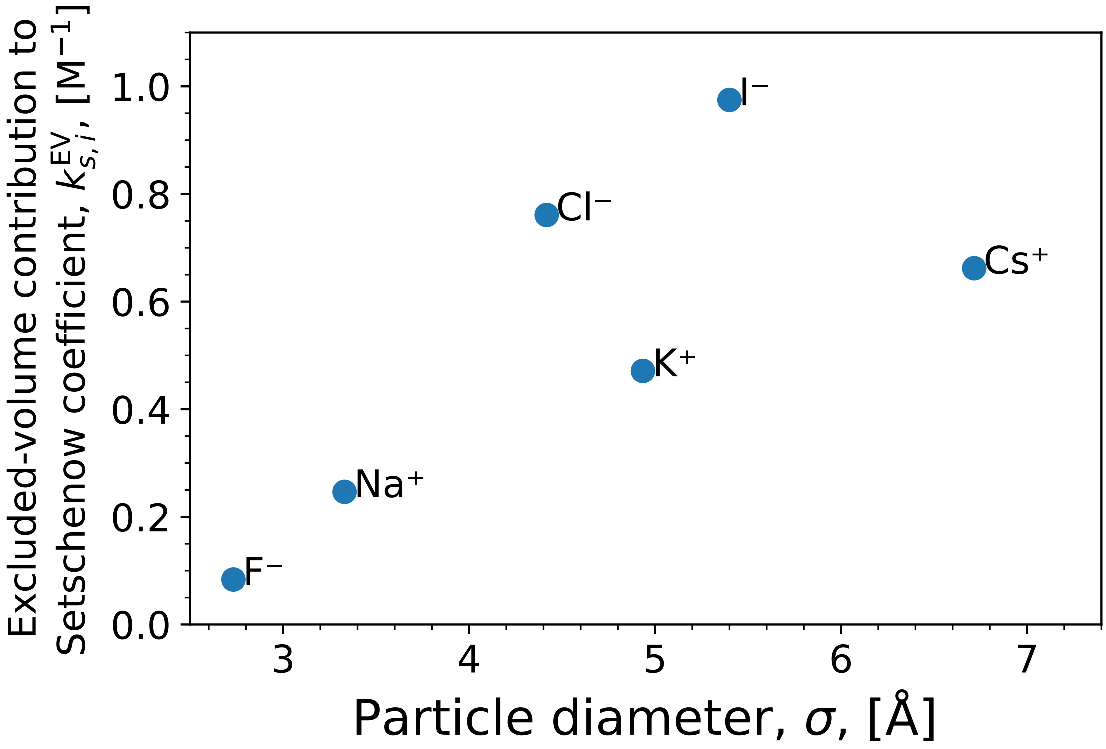

Figure S16: Excluded-volume contribution to the Setschenow coefficient as a function of the particle diameter σ from the Lennard-Jones potential obtained using the OPLS force field. The error bars (standard deviation) are smaller than the symbol size. The linear correlation statistics; R^2: 0.546, two-sided p-value: 0.09322, indicating the correlation for linear correlation is insignificant for the OPLS force field. See the discussion concerning a better correlation variable in section 3.4 in the main text.

17 Supplementary Figure 17: Correlation between the derivative of the water molarity with respect to the salt concentration and the water contribution to the excluded-volume component in the Setschenow coefficient.

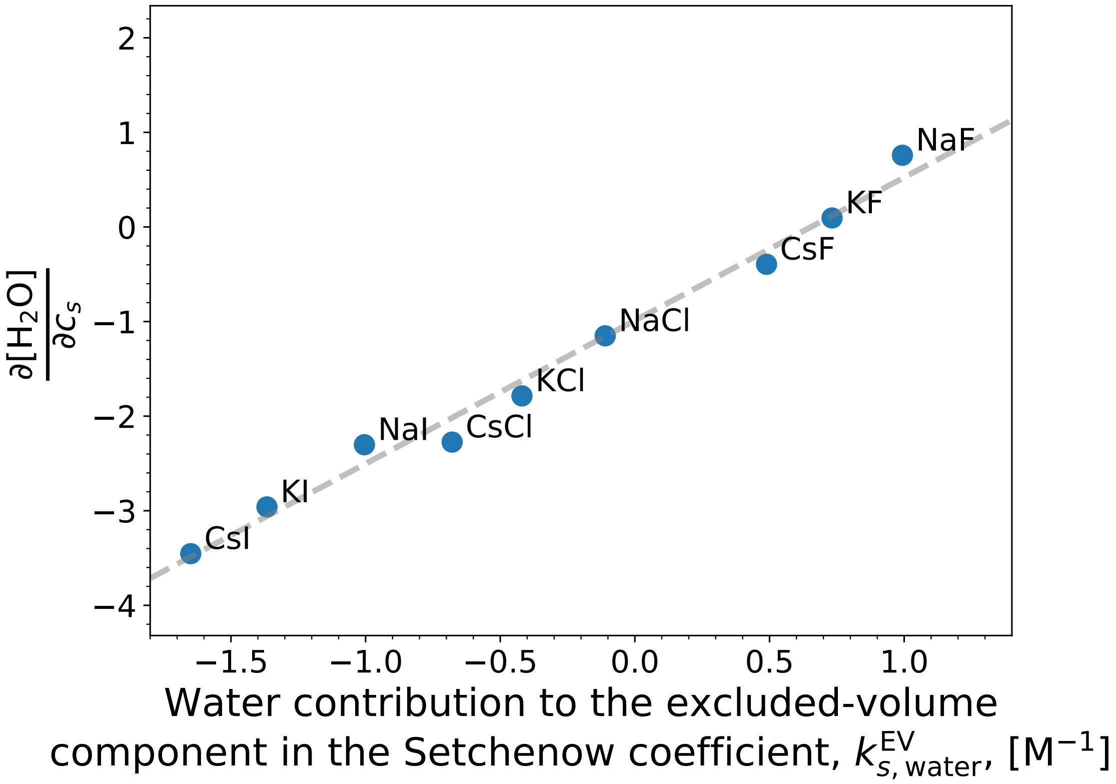

Figure S17: Correlation between the derivative of the water molarity with respect to the salt concentration and the water contribution to the excluded-volume component in the Setschenow coefficient. The data were fitted to a linear equation with the following linear statistics - R^2: 0.987, p-value: 7.66×10^{-08}.

18 Supplementary Figure 18: Correlation between the derivative of the water molarity with respect to the salt concentration and the water contribution to the excluded-volume component in the Setschenow coefficient. (OPLS)

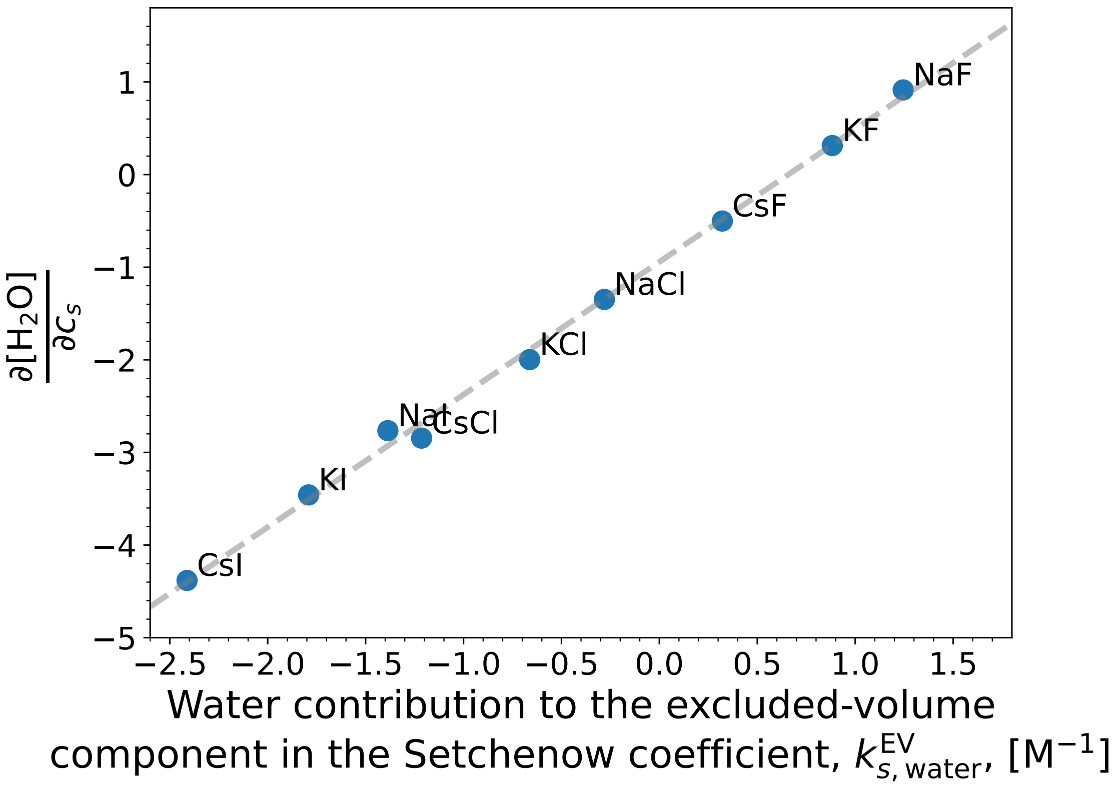

Figure S18: Correlation between the derivative of the water molarity with respect to the salt concentration and the water contribution to the excluded-volume component in the Setschenow coefficient. The data were fitted to a linear equation with the following linear statistics - R^2: 0.997 p-value: 3.894×10^{-10}. The OPLS force field was used here.
